# Association between the traditional Chinese medicine pathological factors of opioid addiction and DRD2/ANKK1 TaqIA polymorphisms

**DOI:** 10.1186/s12906-015-0727-z

**Published:** 2015-07-03

**Authors:** Meimei Cai, Zhiyang Su, Hong Zou, Qin Zhang, Jianying Shen, Lingyuan Zhang, Teng Wang, Zhaoyang Yang, Candong Li

**Affiliations:** Fujian University of Traditional Chinese Medicine, Fuzhou, PR China; Institute of Biomedical Sciences, Fudan University, Shanghai, PR China

**Keywords:** Opioid addicts, Syndrome element, Phlegm syndrome, DRD2/ANKK1 TaqIA, Polymorphism rs1800497,32806C/T

## Abstract

**Background:**

As we known, Traditional Chinese Medicine (TCM) helps to prevent the relapse of drug addiction. However, the scientific basis of TCM remains unclear because of limitations of current reductionist approaches. We aimed to explore the possible mechanism of how ANKK1 TaqIA (A1/A2) [rs1800497(T/C)] affects the relapse of opioid addiction on the perspective of Chinese traditional medicine.

**Methods:**

The ANKK1 TaqIA (A1/A2) [rs1800497(T/C)] of the dopamine D2 receptor (DRD2) polymorphisms were genotyped in a case–control sample consisting of 347 opioid addicts and 155 healthy controls with RT-PCR and the TCM pathological factors were collected by means of Syndrome Elements Differentiation in the case–control sample**.**

**Results:**

DRD2/ANKK1 TaqIA Polymorphisms has no relation with opioid addiction relapse; but for those who were diagnosed with phlegm syndrome, DRD2/ANKK1 TaqIA Polymorphisms affect the replapse of apioid addiction (*P* < 0.05).

**Conclusions:**

DRD2/ANKK1 TaqIA is associated with opioid addict and it is obvious in opioid addicts who suffer from the phlegm syndrome.

## Background

Opioid addiction, a kind of chronic brain disease relapses, which is caused by abuse of heroin, morphine, interacts with the reward system of brain [1]. An interesting hypothesis has arisen from the possible roles of decreased dopamine receptor density resulting in Reward Deficiency Syndrome [2]. Reward Deficiency Syndrome is characterized by a lower basal dopamine level because of insufficient receptor capacity, resulting in a need of a certain amount of dopamine to feel good (this could be achieved by rewarding experiences such as drugs, gambling, alcohol, etc.). Studies on animal models further underline the role of DRD2 in drug addiction as the rewarding effects of opiates were found absent in mice lacking the D2 receptor gene [3, 4]. Genetic polymorphisms of the dopaminergic system include single nucleotide polymorphisms (SNPs) and length polymorphisms of the receptors, transporters and metabolizing enzymes.

The dopamine D2 receptor (DRD2) was characterized previously with *Taq*IA, *Taq*IB, and *Taq*ID SNPs based on restriction digestion of this region with the *Taq*I enzyme. Early studies indicated the A1 allele of *Taq*IA(or the T allele of rs1800497) as a risk factor of substance abuse, alcoholism [5] and heroin dependence [6], but some studies have failed to replicate these findings [7, 8]. Later it turned out that the *Taq*IA restriction fragment length polymorphism (RFLP) is located approximately 10 kilobases downstream from the DRD2 gene, in exon 8 of the ANKK1 (ankyrin repeat and kinase domain containing 1) gene [9], which is a member of the serine/threonine kinase family. The *Taq*IA polymorphism, causing an amino acid change in ANKK1 (Glu713Lys), seems to have a significant effect on the specificity of substrate binding. The protein product of the ANKK1 gene was considered as a negative regulator of the NF-κB (Nuclear Factor-KappaB) transcription factor [10]. Moreover, the expression level of NF-κB-regulated genes was shown to be altered by *Taq*IA variants in an in vitro luciferase system [11]. Since DRD2 is regulated by NF-κB [12, 13], it could be assumed that this ANKK1 variant can indirectly affect DRD2 receptor density. Growing evidence indicated conflicting results about the dopamine receptor D2 (DRD2)/ANKK1)TaqIA single nucleotide polymorphism(rs1800497,32806C/T) and common illicit drug dependence risk including stimulants, opioid and marijuana. In recent years, The meta-analysis suggested that DRD2/ANKK1 TaqIA polymorphism might be associated with opioid dependence risk [14].

But not all the results of study with DRD2/ANKK1 TaqIA and drug addiction are the same. Apart from the reason of gender, race, the individual reflection environment, natural condition may be one of the main reasons, and leads to different results. TCM theory is a holistic view. The doctor of traditional Chinese medicine will consider what you eat, what you do, and how you feel before giving treatment. Carrying out a study by using traditional Chinese medicine methodology, we may discover some different results from previous studies.

Traditional Chinese Medicine has been practiced in China for more than 2000 years, and for the past 200 years, it has been used in treatment of opioid addiction. It is the fact that TCM is effective in controlling opiate withdrawal symptoms and preventing opiate relapse with fewer side effects. In Traditional Chinese Medicine (TCM) theory, the common TCM pathological factors that may induce addiction relapse are yang deficiency, blood heat, and phlegm, among which the most prominent one is phlegm [15], while it is still an unresolved question about how phlegm induces relapse. In order to investigate the association between phlegm and DRD2/ANKK1 TaqIA which plays a key role in relapse, we collected 347 cases for DRD2/ANKK1 TaqIA genotyping, and tried to figure out its relationship with the Traditional Chinese Medicine pathological factors of opioid addiction.

## Materials and methods

### Study object

**Fig. 1:**
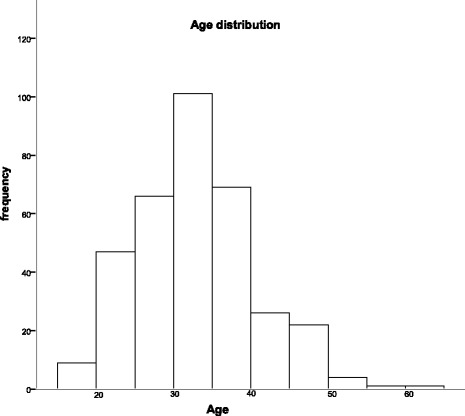
The age distribution of opioid addiction subjects in the present study

**Fig. 2:**
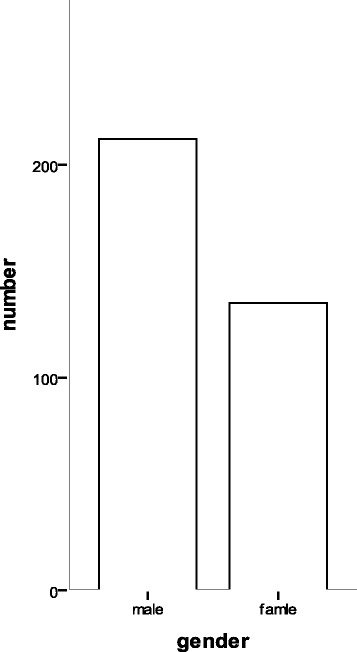
The gender distribution of opioid addiction subjects in the present study

**Fig. 3:**
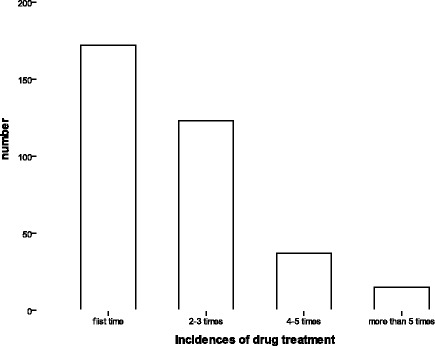
The incidences of drug treatment in opioid addiction subjects

Participants were recruited from November 2008 to December 2012 in Fuzhou City, Fujian Province, China. We recruited 347 opioid addicts from two drug rehabilitation centres (167 subjects came from the drug rehabilitation centers under the jurisdiction of the Department of Public Security of Fujian Province and 180 subjects came from the drug rehabilitation centers under the jurisdiction of the Department of Justice of Fujian Province). The experimental procedures and protocol were approved by the Ethical Committee of Fujian University of Traditional Chinese Medicine. Informed consent forms were signed by all subjects before the study. Figs. 1, 2, and 3 provide the characteristics of the participants, including their age, gender.

All these opioid addiction subjects met the Diagnostic and Statistical Manual of Mental Disorders-IV, followed completion of a 3- month detoxification-treatment, and had no history of mental illness, agreed to participate in this survey. All of them could understand and correctly answer the various assessment tools, and cooperated to complete the questionnaires. Those with liver, kidney, cardiac insufficiency, and those psychopaths who could not cope with the survey were excluded.

**Fig. 4:**
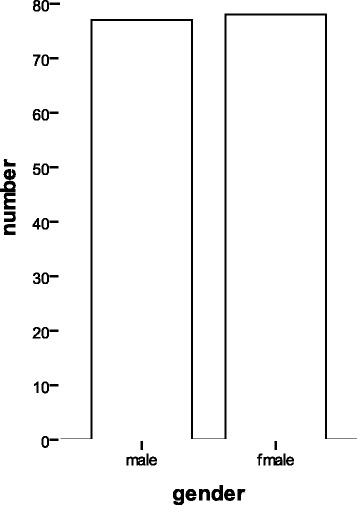
The gender distribution of health group in the present study

The healthy control group: 155 students were randomly selected from schools who were healthy and ranged from 18 to 25 years old without illicit drug addiction, alcohol addiction, smoking and other drug addiction. (Sex ratio of both drug group and health control group had no significant difference, *P* < 0.05) ( Fig. 4).

### Procedure

All subjects participated in a 12-h overnight rapid blood draw. Trained research assistants then completed a survey of physiological characteristics for all participants, and the participants completed questionnaires immediately after breakfast.

### Data collection

#### Syndrome elements differentiation

TCM Syndrome differentiation (also known as ZHENG differentiation) is a process of analyzing data collected through four combined diagnostic methods: inspection, auscultation and olfaction, inquiry, and the palpation is a distinguishing feature of TCM. According to TCM theory, Prof. Wenfeng Zhu established the Syndrome Elements (ZHENG Elements) Differentiation method [16], and it is recognized by Diagnostics of Traditional Chinese Medicine Branch of China Association of Chinese Medicine. This method is used in China to identify TCM syndrome. The criteria of which is set up by statistical means to explore the relationship between TCM syndrome and clinical symptoms of patients. Traditionally, people can be diagnosed with certain TCM syndrome through the four-diagnostic methods, with certain degree of subjectivity and ambiguity from individual doctors. But according to this method, TCM Syndrome differentiation become objectified by statistical means.

What are TCM syndrome elements? For example, if a patient is diagnosed as syndrome “liver Yin deficiency”, according to this method, we could get two TCM syndrome elements: one is Liver ,the other is Yin deficiency. Prof. Zhu Wenfeng calculated the percentage of each symptom appearing in certain TCM syndrome elements through epidemiology studies of large sample, by investigating TCM disease cases books in history and consulting prestigious TCM doctors by Delphi method and then concluded how important a symptom can contribute to certain TCM syndrome element by weighting each symptom with certain integral. Hence, in this method, you can find a certain integral of each symptom which is related to a certain TCM syndrome element. Once the sum of integrals of all symptoms relating to certain syndrome element is equal to or above 100, certain syndrome element is accepted as part of diagnosis for a patient. For example, the patient’s syndrome is yellowing of the skin, (in TCM Theory, skin becoming yellow is relevant to Liver), so it is 40 of symptom “yellowing of the skin”, which contributes to syndrome element “Liver”, according to Wenfeng III Auxiliary Diagnosis and Treat System of TCM [17–19]. Once the sum of integrals of syndrome element is equal to or above 100, certain syndrome element is accepted as part of diagnosis for a patient.

#### Method

*Drug Abuse and Opioid Addiction Severity Inventory (OASI)* [20] and *The Syndrome Differentiation Significance of 600 Kinds of Common Symptom* [16] were used. The drug group’s four kinds of diagnostic information were collected and the simultaneous acquisition was required to achieve the objectivity and accuracy. *Syndrome Elements Differentiation* method was used for reference. Through preliminary experiments, “the TCM syndrome scale for Drug abusers in Fujian province” was designed by trained and professional personnel to investigate objects, item by item.

#### Genotyping

DNA was extracted using standard Methodology. The assay reagents for SNP genotyping consisted of a mix of PCR primers and TaqMan MGB probes (FAM and VIC labeled) that were obtained from Applied Biosystems (Foster City, CA, USA). TaqMan SNP Genotyping Assays (ABI) were used for the polymorphisms ANKK1 rs1800497. Probes and primer for the DRD ANKK1 rs1800497 polymorphism were designed in-house (Probe1: FAMCAC AGCCATCCTCAAAGTGCTGGTCGAGGCAGGCGCCCAGCTGGACGTCCA (5′-3′);Probe2:VICCACAGCCATCCTCAAAGTGCTGGTCAAGGCAGGCGCCCAGCTGGACGTCCA (5′-3′). Each assay enables scoring of both alleles in a single well within a 384-well plate. All assays were optimized to work with genomic DNA and TaqMan Universal Master Mix from Applied Biosystems. Forty cycles of PCR were performed. Plates were analyzed using an ABI Prism 7900HT Sequence Detection System from Applied Biosystems.

### Statistical analysis

The questionnaire data entry was processed by Access 2000 software, and a database was set up, then the calculation of the allele and genotype frequencies and Hardy-Weinberg equilibrium test. Chi-square test was analyzed by SPSS 19.0 software, and statistical analysis of differences in each group allele and genotype frequency distribution was carried out, then came correlation between genotype and syndrome factor using a stepwise logistic regression mode.

## Results

### Relationship between Syndrome Elements and opioid addiction

**Fig. 5:**
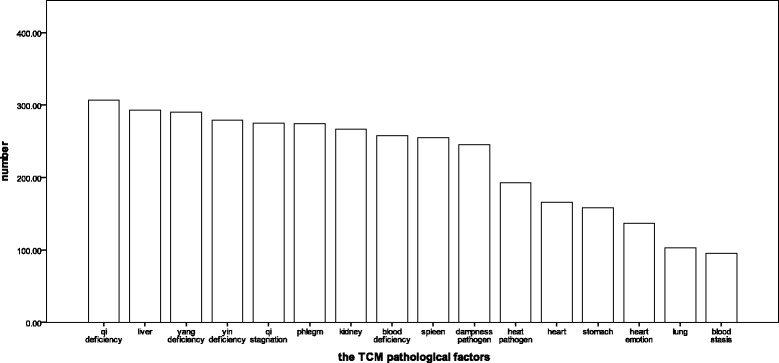
The main Syndrome Elements of opioid addicts

The main Syndrome Elements we collected is shown in Fig. 5, and qi deficiency, liver, yang deficiency are common among opioid addicts. We found the more incidences of drug treatment one person has, the stronger addiction intensity he has, so we weighted “Addiction intensity” as follows:first time =1; more than twice (including 2 times) = 2. The age, gender and duration of drug use (years) had no difference in opioid addicts who have different addiction intensity. A stepwise logistic regression mode was used when age and gender were always included to analyze the relationship between Syndrome Elements and opioid addiction. In the mode, the integral of each TCM syndrome element is the independent variable, and addiction intensity is the dependent variable. The stronger the addiction intensity is, the more possible the relapse will come.

As shown in Table 1, phlegm, qi deficiency, damp evil, blood stasis, lung and heart emotion are significantly associated with addiction relapse, comprising phlegm qi deficiency (*P* = 0.000, 95 % CI:,0.708 to 0.962). This shows that people with phlegm syndrome element (*P* = 0.000, 95 % CI:1.131 to1.287) or qi deficiency syndrome element(*P* = 0.001, 95 % CI:1.021 to 1.051) have stronger addiction intensity and are more likely to relapse. Damp evil(*P* = 0.000, 95 % CI:0.915 to0.966), blood stasis(*P* = 0.008, 95 % CI:0.954 to0.988), lung(*P* = 0.000, 95 % CI:0.823 to0.930) and heart emotion(*P* = 0.000, 95 % CI:0.883 to0.960) are the protective factors of addiction relapse. According to the result, phlegm, qi deficiency, damp evil, blood stasis, lung and heart emotion are the TCM pathological factors which affect the relapse of opioid addiction.

**Table 1:** Relationship between Syndrome Elements and opioid addiction

Participant characteristics	P	OR	CI(95 %)
Age	0.104	1.089	0.983-1.207
Gender	0.444	1.813	0.396-8.312
Phlegm	0.000	1.206	1.131-1.287
Damp evil	0.000	0.940	0.915-0.966
Blood stasis	0.008	0.954	0.921-0.988
Qi deficiency	0.001	1.031	1.012-1.051
Lung	0.000	0.875	0.823-0.930
Heart emotion	0.000	0.920	0.883-0.960

### DRD2/ANKK1 TaqIA Distribution

DRD2/ANKK1 TaqIA genotype and allele frequency distribution are shown in Table 2. The distribution of the DRD2/ANKK1 TaqIA was in Hardy-Weinberg (HW) equilibrium (*P* > 0.05), and the distribution did not differ between the females and males, the opioid addicts and the health group, respectively. To further determine differential distribution in opioid addicts, we carried out Pearson Chi-square Test. We find there is no difference in opioid addicts who have different Addiction intensities (Table 2).

**Table 2:** DRD2 TaqIA genotypic distribution and allelic frequencies

	Genotypic Distribution (%)			Allele Frequency (%)		
Group	A1A1	A1A2	A2A2	A1	A2	HWE*P*-value
Full (*n* = 502)	71 (14.1)	252 (50.2)	179 (35.7)	394 (39.2)	610 (60.8)	0.238
Males (*n* = 289)	39 (13.5)	137 (47.4)	113 (39.1)	215 (37.2)	363 (62.8)	
Females (*n* = 213)	32 (15.0)	115 (54.0)	66 (31.0)	179 (42.0)	247 (58.0)	
Opioid addicts (*n* = 347)	50 (14.4)	168 (48.4)	129 (37.2)	268 (38.6)	426 (61.4)	0.692
Health group (*n* = 155)	21 (13.5)	84 (54.2)	50 (32.3)	126 (40.6)	184 (59.4)	0.125
Addiction intensity = 1	26 (7.49)	88 (23.4)	58 (16.7)	140 (20.2)	204 (29.4)	
Addiction intensity = 2	24 (6.91)	81 (23.3)	70 (20.2)	129 (18.6)	221 (31.8)	

Genotype comparison between males and females: χ^2^ = 3.53, df = 2, *p* = 0.171

Genotype comparison between opioid addicts and health group: χ^2^ = 1.034, df = 2, *p* = 0.596

Genotype comparison between different addiction intensities: χ^2^ = 1.469, df = 2, *p* = 0.480

Full: includes both males and females

Hardy-Weinberg equilibrium of genotype distributions of each polymorphism was tested for all of the participants, opioid addicts, and health group

### Relationship between TCM pathological factors of opioid addiction and DRD2/ANKK1 TaqIA

As we known, the distribution of DRD2/ANKK1 TaqIA has no difference in opioid addicts who have different addiction intensities, and we find there is no relationship between the DRD2/ANKK1 TaqIA and Syndrome Elements by using one-way analysis of variance (Table 3). Furthermore, we applied a stepwise logistic regression model to DRD2/ANKK1 TaqIA, Syndrome Elements and Addiction intensity to explore the relevance between them. As shown in Table 4, the genotype of DRD2/ANKK1 TaqIA has no relationship with addiction intensity, and if excluded from the model, the interaction of DRD2/ANKK1 TaqIA and phlegm is an important factor in the addiction intensity. The DRD2/ANKK1 TaqIA genotype(A2A2) could add addiction intensity (*P* = 0.007, 95 % CI:0.995 to1.021) relative to TaqIA genotype(A1A1). The influence of the heterozygote(A1A2) was he same with genotype(A1A1), so we could find A2 is an risk factor of addiction relapse, when phlegm exists.

**Table 3:** Relationship between the DRD2/ANKK1 TaqIA and Syndrome Elements

Syndrome Elements	F	P
Qi deficiency	0.048	0.953
Liver	0.397	0.692
Yang deficiency	0.077	0.926
Yin deficiency	0.367	0.693
Qi stagnation	0.206	0.814
Phlegm	0.430	0.651
Kidney	0.016	0.984
Blood deficiency	0.004	0.996
Spleen	0.031	0.970
Dampness pathogen	0.236	0.790
Heat pathogen	0.492	0.612
Heart	0.208	0.813
Stomach	0.466	0.628
Heart emotion	1.157	0.316
Lung	0.215	0.807
Blood stasis	0.032	0.968

**Table 4:** Relationship between TCM pathological factors of opioid addiction and DRD2/ANKK1 TaqIA

Participant characteristics	P	OR	CI(95 %)
age	0.424	1.043	0.941-1.154
gender	0.274	2.679	0.458-15.668
phlegm	0.000	1.305	1.159-1.470
damp evil	0.000	0.914	0.872-0.986
blood stasis	0.010	0.941	0.889-0.986
qi deficiency	0.002	1.048	1.017-1.080
lung	0.000	0.818	0.740-0.914
heart emotion	0.000	0.870	0.808-0.937
DRD2/ANKK1TaqIA * phlegm	0.014		
DRD2/ANKK1TaqIA(A2A1) * phlegm	0.225	1.008	0.995-1.021
DRD2/ANKK1TaqIA(A2A2) * phlegm	0.007	1.027	0.995-1.021

## Discussion

Relapse is a complex pathological behavior, caused by the combination of the pathological psychological, pathophysiological and social environmental factors [21]. Besides genetics, age, sex, diet, spirit endures, it is widely believed in China that Zang-Fu function, Qi-blood and Body fluids change in TCM theory have association with drug relapse behavior. While currently, relevant studies are scarce.

TCM is a holistic and systematic medicine, and Syndrome Elements Differentiation method based on TCM Syndrome Differentiation was used to extract TCM pathological factors. Our statistics results suggest that in both group1 and group2, phlegm is a risk factor for addiction relapse. And qi stagnate, damp are protection factors, which is consistent with our previous findings [15].

Dopamine receptors belong to the G protein coupled receptor family. Studies have shown that DRD2 gene knockout mice had failed to react to opioids reward [22], showing that DRD2 plays an important role in drug addiction. DRD2 gene is located in human genome 11q22 near the outer 8 exon 3′- untranslated region which contains a TaqIA polymorphism. Among them there are A1, A2 allele. The correlation between the above site and drug addiction has previously been reported [23–25]. In our study, the results indicate that the distribution of genotypes and allele frequencies between the drug group and the normal control group showed no significant difference (*P* > 0.05), which is contrary to the results of previous studies. The reasons we assume are as follows: first, there are varying gene frequency distributions among different geographic regions; second, different drugs using and short-term drug dependence might deviate the experiment result.

Long-term opioid abuse caused Qi-blood disturbance, and Qi and blood were consumed, then leading to dysfunction of the zang-fu organs, and producing all sorts of Traditional Chinese Medicine pathological factors. The performance of opioid addicts show that their oral secretions increased, be easy to fatigue, in the bad emotional state, and so on, which is related with phlegm, qi deficiency, damp evil, and anther Traditional Chinese Medicine pathological factors, especially phlegm. Yawning, runny nose, tears, and frequently increasing oral secretions arose during Drug addiction episodes. That is the same with the performance of phlegm syndrome. And the previous data mining research on the relationship between the loci and addiction relapse shows once phlegm symptom was present, the addiction of patients who carry the A2A2 allele have more possibilities to relapse than those with genotype A1A1. According to our former research, phlegm syndrome is a major cause of addiction relapse, but the mechanism about how phlegm is involved is still a question. From the results of the current data, we found A2A2 had no effect on addiction relapse when TCM factors are not involved. However when DRD2/ANKK1 TaqIA stay with phlegm, it becomes an important factor for the addiction relapse. And A2A2 is a risk factor for addiction relapse. According to the above results we explain as follows: first, phlegm causes addiction relapse, but has no relation with the genotype. The other explanation is phlegm syndrome may affect the expression by modifying the allele through epigenetic mechanisms, thus leading to relapse. In modern research, investigators found opioid receptor gene (OPPM1) was highly methylated in the blood and semen of patients with opioid addiction [26]. This indicates long-term drug dependence is related to epigenetic. The contemporary epigenetic theory researchers believe that at every stage of life, genome and epigenome damage are associated with the harmful health factors. When exposed to the a particular environment, defect metabolic process or improper supply of trace elements are involved in DNA synthesis, DNA damage repair, and DNA normal methylation process. Currently, more and more studies show phlegm has close relationship with energy metabolism and the immune system, and it could lead to form a particular environment in the body that is conducive to epigenetic. And in that situation, charming away phlegm will help addicts to recover from drug addiction. As in the related researches, An-jun-ning, a traditional herbal formula that could reduce phlegm, may help to prevent relapse in opioid dependence treatment. Studies find AJN can effectively alleviate opioid withdrawal symptoms and preserve or restore the DAT, D2R, TH levels in the striatum. The mechanism underlying the effect of AJN on withdrawal symptoms may be related to the modulation of the dopamine system by AJN [27]. Consequently, we hypothesized that the normal DNA methylation is disturbed when the sputum symptom starts to interfere with the metabolic process.

In this research, we also found other TCM pathological factors associate with addiction relapse, and phlegm is not an independent risk factor. But in this paper; we discussed phlegm syndrome only, the mechanism of the other TCM pathological factors is still unclear.

## Conclusion

DRD2/ANKK1 TaqIA polymorphism is associated with opioid dependence risk, when individual is diagnosed with phlegm syndrome. We suspect that plegm syndrome could lead to form a particular environment in the body that is conducive to epigenetics of DRD2/ANKK1 TaqIA, further caused the addiction relapse. Further studies are needed to discover the specific mechanism. Epigenetic experiment is also necessary.

## Abbreviations

TCM: Traditional chinese medicine
DRD2: Dopamine D2 Receptor
ANKK1: Ankyrin repeat and kinase domain containing 1
LTL: Leukocyte telomere lengths
